# A sex-biased imbalance between Tfr, Tph, and atypical B cells determines antibody responses in COVID-19 patients

**DOI:** 10.1073/pnas.2217902120

**Published:** 2023-01-20

**Authors:** Jonas Nørskov Søndergaard, Janyerkye Tulyeu, Ryuya Edahiro, Yuya Shirai, Yuta Yamaguchi, Teruaki Murakami, Takayoshi Morita, Yasuhiro Kato, Haruhiko Hirata, Yoshito Takeda, Daisuke Okuzaki, Shimon Sakaguchi, Atsushi Kumanogoh, Yukinori Okada, James Badger Wing

**Affiliations:** ^a^Human Immunology Team, Center for Infectious Disease Education and Research, Osaka University, Suita 565-0871, Japan; ^b^Department of Respiratory Medicine and Clinical Immunology, Osaka University Graduate School of Medicine, Suita 565-0871, Japan; ^c^Department of Statistical Genetics, Osaka University Graduate School of Medicine, Suita 565-0871, Japan; ^d^Department of Immunopathology, World Premier International Immunology Frontier Research Center (WPI-IFReC), Osaka University, Suita 565-0871, Japan; ^e^Laboratory of Human Immunology (Single Cell Genomics), WPI-IFReC, Osaka University, Suita 565-0871, Japan; ^f^Genome Information Research Center, Research Institute for Microbial Diseases, Osaka University, Suita 565-0871, Japan; ^g^Integrated Frontier Research for Medical Science Division, Institute for Open and Transdisciplinary Research Initiatives, Osaka University, Suita 565-0871, Japan; ^h^CiDER, Osaka University, Suita 565-0871, Japan; ^i^Laboratory of Experimental Immunology, WPI-IFReC, Osaka University, Suita 565-0871, Japan; ^j^Department of Experimental Pathology, Institute for Frontier Medical Sciences, Kyoto University, Kyoto 606-8507, Japan; ^k^Laboratory of Statistical Immunology, WPI-IFReC, Osaka University, Suita 565-0871, Japan; ^l^Integrated Frontier Research for Medical Science Division, Institute for Open and Transdisciplinary Research Initiatives, Osaka University, Suita 565-0871, Japan; ^m^Laboratory for Systems Genetics, RIKEN Center for Integrative Medical Sciences, Yokohama 230-0045, Japan; ^n^Department of Genome Informatics, Graduate School of Medicine, the University of Tokyo, Tokyo 113-0033, Japan; ^o^Laboratory of Human Single Cell Immunology, WPI-IFReC, Osaka University, Suita 565-0871, Japan

**Keywords:** T-peripheral helper (Tph), T-follicular regulatory (Tfr), atypical B cells, COVID-19, mass cytometry

## Abstract

Sex-biased humoral immune responses to COVID-19 patients have been observed, but the cellular basis for this is not understood. Using single-cell proteomics by mass cytometry, we find disrupted regulation of humoral immunity in COVID-19 patients, with a sex-biased loss of circulating follicular regulatory T cells (cTfr) at a significantly greater rate in male patients. In addition, a male sex-associated cellular network of T-peripheral helper, plasma blasts, proliferating and extrafollicular/atypical CD11c^+^ memory B cells was strongly positively correlated with neutralizing antibody concentrations and negatively correlated with cTfr frequency. These results suggest that sex-specific differences to the balance of cTfr and a network of extrafollicular antibody production-associated cell types may be a key factor in the altered humoral immune responses between male and female COVID-19 patients.

## Significance

A major gap in our current knowledge of COVID-19 is our limited understanding of the pathways of antibody production during acute disease. While a key pathway of antibody production driven by T-follicular helper cells is inhibited, neutralizing antibodies are still produced but may be of variable quality and specificity. Here, we describe that antibody concentrations, in the serum of severe COVID-19 patients, are closely associated with a network of extrafollicular T and B cells, while T-follicular regulatory cells—a key population responsible for supressing antibody production—are reduced most strongly in male patients. As such, these findings clarify the complex network of cells responsible for antibody production in COVID-19 patients.

Since the initial outbreak in late 2019 (1), the devastating SARS-CoV-2 pandemic and the associated COVID-19 disease have had a severe impact on the global community. Susceptibility to infection appears to be driven by a range of factors, with risk increasing particularly with age and male sex (2). Understanding the changes to the immune system of infected patients is imperative since both viral clearance and many acute symptoms are mediated by the immune system (3, 4). Several previous studies have analyzed the immune response in COVID-19 patients and demonstrated significant dysregulation of almost every immune population (5–8). This is also true for regulatory T cells (Tregs), with several groups having reported some degree of disruption in their frequency, although a clear consensus has yet to emerge (9, 10).

Foxp3-expressing Tregs play a key role in the control of the immune system due to their ability to suppress the function of a wide range of cell types and prevent severe autoimmunity (11). Tregs are also known to dampen the resolution phase of an infection and have been demonstrated to have an important role in the response to various infectious diseases such as influenza and malaria (12, 13). Of particular relevance in the context of viral lung infections are the role of the specialized Treg subset T follicular regulatory T cells (Tfr) in controlling plasma cell formation, the quality of the specific antibodies, emergence of autoreactive antibodies, B cell memory, and protection from lung damage during influenza infection (14–16). Several recent reports (17, 18) have demonstrated that many patients with COVID-19 produce autoantibodies, which may have a critical role in the progress of infection due to their ability to neutralize protective host factors such as interferons. In some cases, these autoantibodies are a preexisting risk factor prior to infection. However, there is also clear evidence of de novo generation (19). These factors suggest that Tregs and Tfr may be an important factor in understanding both susceptibility to, and recovery from, COVID-19.

Considering these prior findings, we hypothesized that Tregs may have potential roles in acute antiviral response in COVID-19 patients. In this report, we leverage the ability of single-cell proteomics (mass cytometry) to resolve rare populations, such as Treg subsets, while also retaining a broad view of the immune system in a large patient cohort. We find that subsets of Tregs are key parts of the changing cellular networks related to the severity and sex of patients. Most notably, we see that patients with COVID-19 have a reduced ratio of circulating (c)Tfr to a network of antibody production-associated cells such as T-peripheral helper (Tph), plasma blasts, and CD11c^+^CXCR5^−^ extrafollicular/atypical B cells, which in turn is strongly correlated with neutralizing antibody levels in the serum. Significant sex bias was also seen, with cTfr being highest in female sex and the extrafollicular cell network associated with male sex. Our data provide cellular evidence of dysregulated antibody responses, which could explain previous reports (18, 20–22) of increased antibody responses in male COVID-19 patients.

## Results

### COVID-19 Generates Atypical CTLA-4^high^ Effector and CXCR4^high^ Naïve Conventional CD4+ T Cells.

**Fig. 1.**
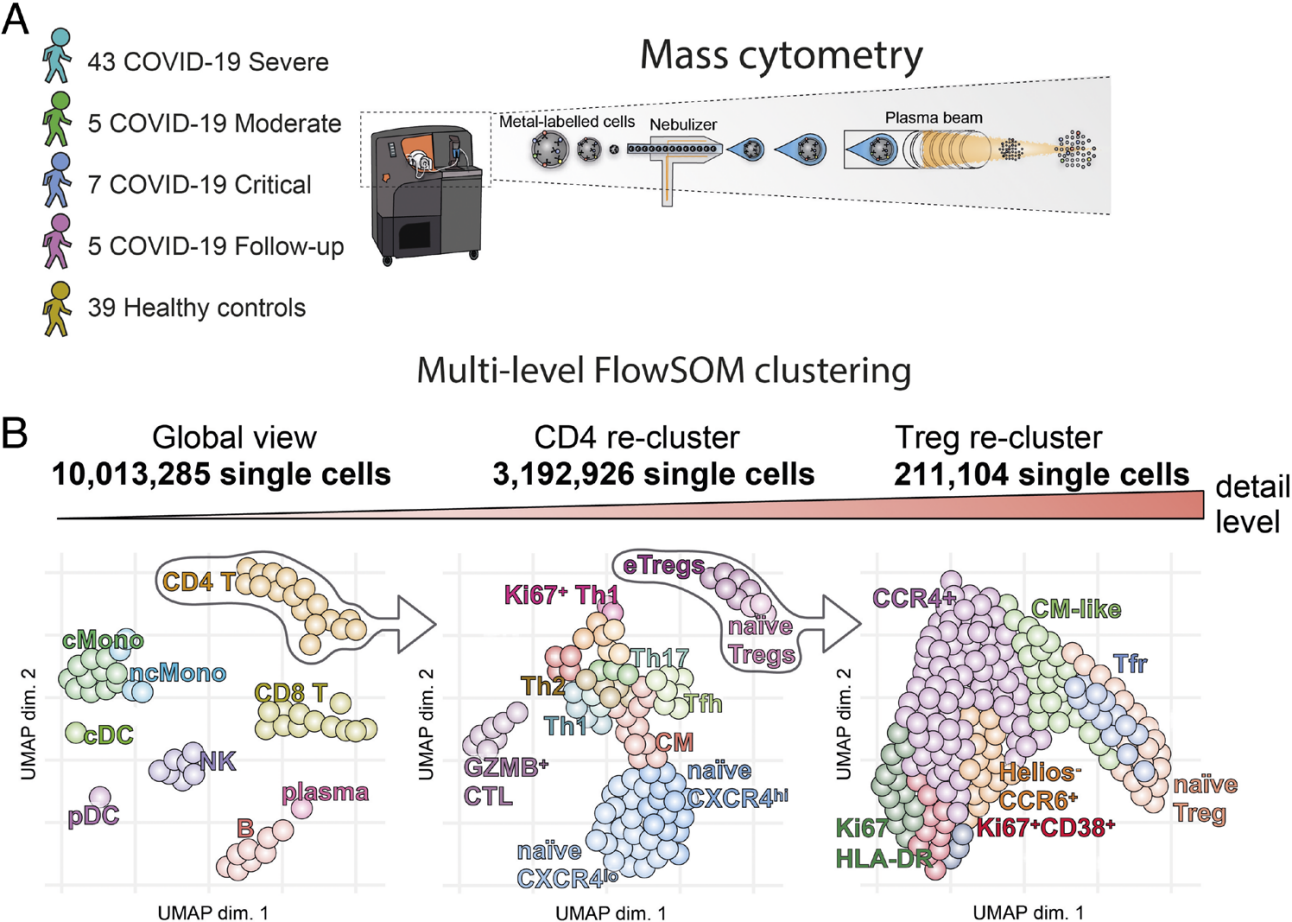
Schematic diagrams of study. (*A*) Patient cohort and mass cytometry schematic. (*B*) Multilevel FlowSOM analysis schematic.

Peripheral blood mononuclear cells (PBMCs) from 40 healthy controls (HCs) and 55 COVID-19 patients (Table 1) were labeled with metal-tagged antibodies and analyzed on a Helios mass cytometer (Fig. 1). Self-organizing map (FlowSOM) clustering of CD45^+^ live cells showed clear resolution of most major immune subsets (*SI Appendix*, Fig. S1*A*). Analysis of changes to cellular frequency demonstrated that in comparison to HCs, severe COVID-19 patients had significantly increased frequencies of B cells, plasma blast cells (plasma), and classical monocytes (cMono) (*SI Appendix*, Fig. S1 *B* and *C*). In contrast, CD8 T cells, nonclassical monocytes (ncMono), conventional dendritic cells (cDCs), and plasmacytoid dendritic cells (pDCs) were all significantly reduced in a manner similar to those of previous reports (7, 8). Moderate and critical patients’ immune composition was comparable to that of severe patients at this global level with the exception that moderate patients retained a more normal proportion of CD8 T cells (*SI Appendix*, Fig. S1 *B* and *C*). Follow-up samples had mostly returned to similar proportions as HCs, indicating that these cellular changes were transient. To obtain a fine resolution of cellular populations, we then performed a subset analysis of major cell types: CD4 T cells (CD4), CD8 T cells (CD8), NK cells (NK), B and plasma cells (B cells, plasma), and myeloid cells and DCs (pDC, cDC, ncMono, cMono). Low frequencies of cell doublets and PBMC contaminating neutrophils were excluded from further analysis at this stage.

**Table 1.** Patient information

	HCs < 50	HCs 50+	Moderate	Severe	Critical	Follow-up
Number	24	15	5	43	7	5
Mean age (min-max)	33.7 (22−47)	54.5 (50−64)	56.6 (39−78)	67.5 (28−86)	64.7 (59−73)	72 (59−80)
Female/total	7/24	11/15	1/5	17/43	1/7	1/5
Mean male age/female age	N/D	N/D	61/39	66/69	64.8/64	72/72
Mean BMI (min-max)	N/D	N/D	25 (15.5−34.7)	24.4 (18.3−38)	29.506 (22−35.9)	21.99
BMI measured/total	0/24	0/15	5/5	36/43	5/7	4/5
Mean male BMI/female BMI	N/D	N/D	27.41/15.5	24.1/24.9	31.3/22	21.99/ND
Diabetes mellitus type 2/total	N/D	N/D	0/5	15/43	4/7	2/5
COPD/total	N/D	N/D	0/5	3/42	1/7	1/5
Hypertension/total	N/D	N/D	1/5	18/43	2/7	2/5
Asthma/total	N/D	N/D	0/5	1/43	0/7	0/5
Hashimoto disease/total	N/D	N/D	0/5	1/43	0/7	0/5
Emphysema/total	N/D	N/D	0/5	1/43	0/7	0/5
Rheumatoid arthritis (no treatment)/total	N/D	N/D	0/5	1/43	0/7	0/5
Chronic kidney disease (CKD)/total	N/D	N/D	0/5	4/43	0/7	0/5
Systemic lupus erythematosus (SLE)	N/D	N/D	0/5	0/43	0/7	0/5
Mean days after symptom onset (min-max)	N/A	N/A	7.6 (6−9)	10.5 (6−18)	14 (6−25)	62.4 (56−72)
Mean days after symptom onset (male/female)	N/A	N/A	8/6	11.5/9.2	14/6	62.5/64
Mean days after intubation/intubated at the time of sampling	N/A	N/A	N/A (0/5)	2.02 (42/43)	3.83 (6/7)	50.75 (2/5)
Mean days after starting steroids (min-max)	N/A	N/A	1.6 (1−2)	4.53 (1−13)	7.14 (1−19)	55.5 (52−62)
Steroid treated/total	N/A	N/A	5/5	43/43	7/7	5/5
Tocilizumab treated/total	N/A	N/A	0/5	2/43	2/7	0/5

**Fig. 2.**
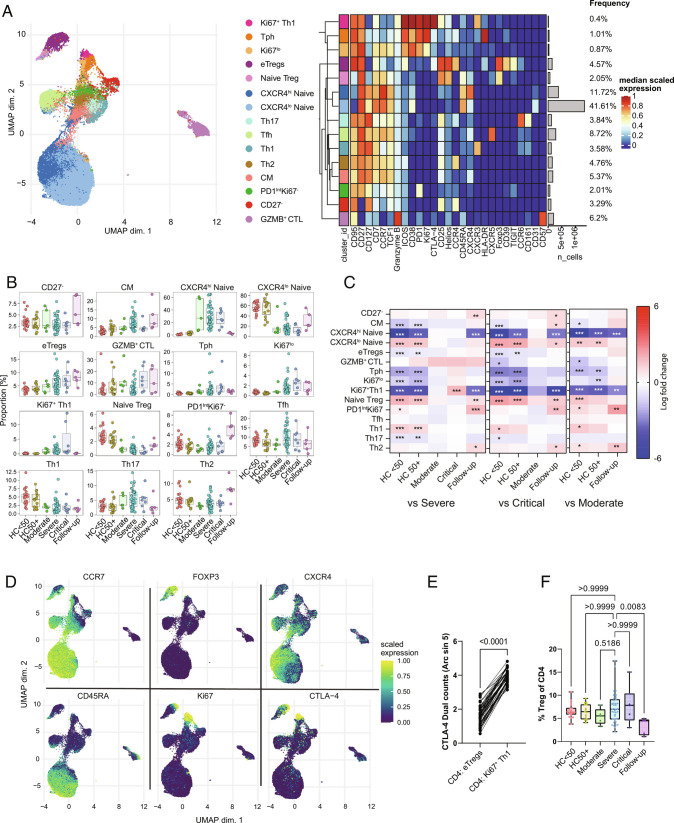
CD4 T cell phenotypes in COVID-19. (*A*) UMAP and expression heatmap of FlowSOM clusters from CD4 T cells. The heatmap displays scaled expression of indicated markers. (*B*) Frequency boxplots of proportion within the CD4^+^ T cell cluster of cells. HCs under 50 y of age (HC < 50), HCs of 50 y of age or above (HC50+), moderate, severe, critical, or follow-up COVID-19 patients. (*C*) Comparison of the fold change (log_2_) in cluster frequency between the indicated group and severe (*Left*), critical (*Middle*), and moderate (*Right*) COVID-19 patients. (*D*) UMAP feature plots of selected markers. (*E*) Expression of CTLA4 on eTregs vs. Ki67^+^ Th1 cells. (*F*) Percentage of Tregs as a proportion of total CD4 T cells. *P ≤ 0.05, **P ≤ 0.01, ***P ≤ 0.001 or value indicated on the graph. Significance by edgeR (*C*), Wilcoxon matched-pairs (*E*) or Kruskal–Wallis (*F*). Effector Treg (eTreg), CM, granzyme B-positive cytotoxic lymphocyte (GZMB^+^ CTL). Expression values are arcsinh-transformed (cofactor: 5) dual counts.

In-depth subclustering of CD4 T cells identified a range of previously described CD4 T cell populations that we manually annotated based on an examination of uniform manifold approximation and projection (UMAP) distribution and expression of markers used for clustering (Fig. 2*A*). For example, central memory (CM) cells were identified as CD95^+^CCR7^+^CD45RA^–^ cells located between the naïve and effector memory (EM) areas of the UMAP, while granzyme B^+^ cytotoxic CD4 T cells (annotated as GZMB^+^ CTL) were identified as CD95^+^CD57^+^GZMB^+^ (Fig. 2*A*). Changes to the proportions of clusters in COVID-19 revealed perturbations across the spectrum of naïve to effector cells (Fig. 2 *B* and *C*). Expansion of several groups of proliferative Ki67^+^ CD4 cells was seen including a group of less differentiated Ki67^lo^ cells that retained TCF1 and CCR7 (annotated as Ki67^lo^) and two more terminally differentiated HLA-DR^+^ (HLA-DR^+^Ki67^+^) and CXCR3^+^ (Ki67^+^Th1) subgroups mostly lacking markers of stemness such as TCF1 (23). Nonproliferating CXCR3^+^ cells (Th1) were reduced in COVID-19 patients, potentially as they had shifted to the Ki67^+^Th1 group (Fig. 2*B*). Interestingly, we observed that the Ki67^+^Th1 cluster was significantly higher in critical than that of severe patients (Fig. 2 *B* and *C*) and showed extremely high levels of intracellular CTLA-4 (Fig. 2 *A* and *D*). While CTLA-4 is not exclusively expressed by Tregs, in both healthy donor PBMCs and most highly activated environments such as tumors, effector Tregs reliably express higher levels of CTLA-4 than all other populations of effector CD4 and CD8 (24). However, in this case, the Ki67^+^Th1 cluster had significantly higher CTLA-4 expression than that of even effector Tregs (Fig. 2*E*). To better understand the relationship between these CTLA-4^hi^ cells and other effector groups, we used trajectory analysis. The COVID-19-enriched effector CD4 T cell population had a separate trajectory from other effector CD4 T cells, rather running in parallel to Tregs due to their relative phenotypic similarity (*SI Appendix*, Fig.S2 *A* and *B*). However, despite high CTLA-4, the Ki67^+^Th1 subgroup had a much lower level of Foxp3 expression than naïve Tregs, suggesting that the Ki67^+^Th1 cluster can be considered as Foxp3^lo/–^ non-Tregs (25).

A significant shift in clusters within phenotypically naïve CD4 cells was also observed in COVID-19 patients. This was primarily characterized by increased expression of CXCR4, the chemokine receptor for CXCL12, and a resulting shift in the frequency of clusters from CXCR4^lo^ naïve to CXCR4^hi^ naïve in moderate, severe, and critical COVID-19 patients and then returned to CXCR4^lo^ naïve in the follow-up samples (Fig. 2 *B*–*D*). These CXCR4^hi^ naïve cells retained the expression of CD45RA, CD27, CD127, TCF1, and CCR7, but close examination indicated slight changes to the expression of some markers (Fig. 2*A*), suggesting a later stage of development. To confirm this, we verified that the CXCR4^hi^ naïve cluster had significantly reduced expression of the recent thymic emigrant marker CD31 (*SI Appendix*, Fig. S2*C*). Significant but low upregulation of CD95 was also seen (*SI Appendix*, Fig. S2*D*), suggesting a phenotypic similarity with CD95^+^CD45RA^+^ T-stem memory cells (26). However, since most naïve cells in COVID-19 patients gain this phenotype, we consider it unlikely that this is driven by antigen-specific memory development, but rather that CXCR4 may prime naïve T cells for trafficking to the lungs of COVID-19 patients (27).

### Disruption of Tregs in COVID-19.

**Fig. 3.**
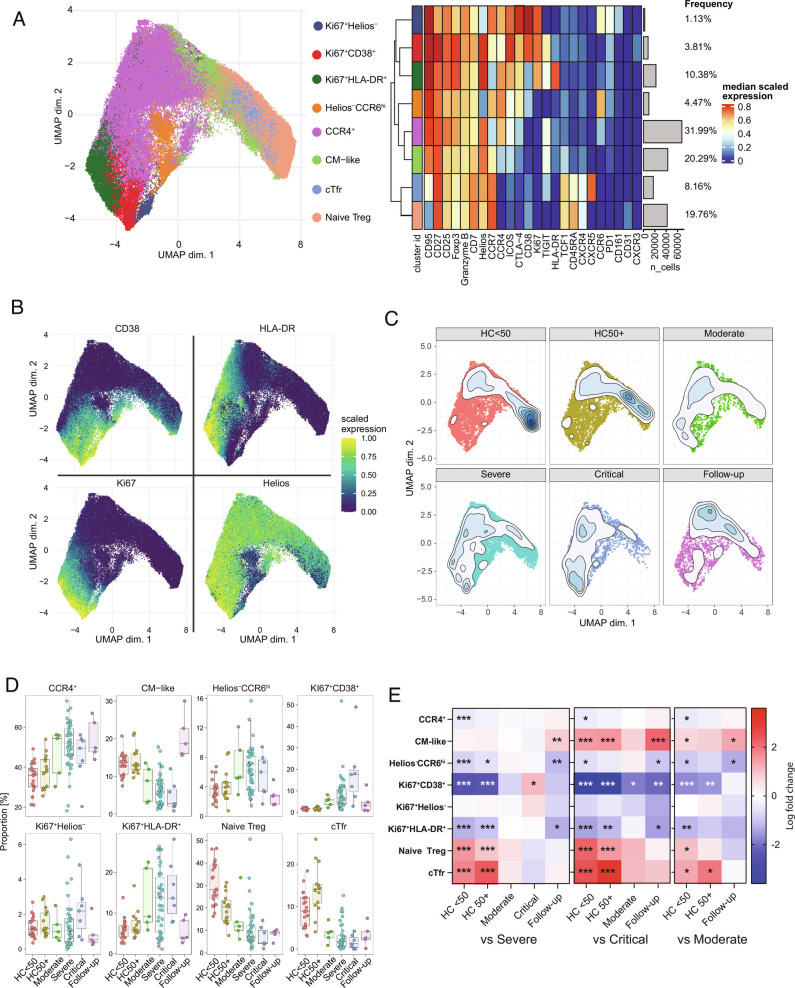
Treg phenotypes in COVID-19. (*A*) UMAP and expression heatmap of FlowSOM clusters from subclustering of Treg cells. (*B*) Scaled expression of indicated markers displayed on the UMAP. (*C*) Population density of cells displayed on UMAP. (*D*) Frequency boxplots of proportion of Treg cells from indicated clusters. HCs under 50 y of age (HC < 50), HCs of 50 y of age or above (HC50+), moderate, severe, critical, or follow-up COVID-19 patients. (*E*) Comparison of the fold change (log_2_) in Treg cluster frequency between the indicated group and severe (*Left*), critical (*Middle*), and moderate (*Right*) COVID-19 patients. *P ≤ 0.05, **P ≤ 0.01, ***P ≤ 0.001 by edgeR. Expression values are arcsinh-transformed (cofactor: 5) dual counts.

Several reports on COVID-19 have demonstrated some degree of disruption in Tregs (9). We saw that Tregs as a proportion of CD4 were not changed in acute COVID-19 patients but appeared to be reduced at the follow-up stage (Fig. 2*F*). Additionally, some shift from naïve to effector Tregs was clear at the CD4 level (Fig. 2 *A* and *B*). Tregs are a complex population with a range of known subtypes (28). To gain a better resolution of their subphenotypes, we performed subclustering (Fig. 3*A*). While there is no true consensus of Treg subpopulations, we were able to recapitulate most described Treg subpopulations such as naïve, Tfr, an intermediate CM-like population, CCR4^+^ effectors, Helios^–^CCR6^+^ cells, and several groups of highly activated HLA-DR or CD38-expressing Tregs (25, 29–34) (Fig. 3 *A* and *B*). A shift toward activated Treg subtypes was seen in patients with COVID-19 with increases in the activated CCR4^+^ (annotated as CCR4^+^), Helios^–^CCR6^+^ effectors, and several groups of proliferating Tregs (Fig. 3 *C*–*E*). A corresponding reduction in the proportions of naïve, Tfr, and CM-like population was observed. The proliferating Helios^–^ cluster (Ki67^+^Helios^–^) lacked any clear association with COVID-19, while the Ki67^+^HLA-DR^+^ cluster was generally increased in all COVID-19 patient groups. However, the CD38^hi^HLA-DR^–^ group of proliferating Tregs (Ki67^+^CD38^+^) was significantly increased in critical patients in comparison to severe or moderate groups (Fig. 3 *D* and *E*), suggesting an association with the most severe forms of the disease.

### Treg Subsets Are Central Hubs in COVID-19.

**Fig. 4.**
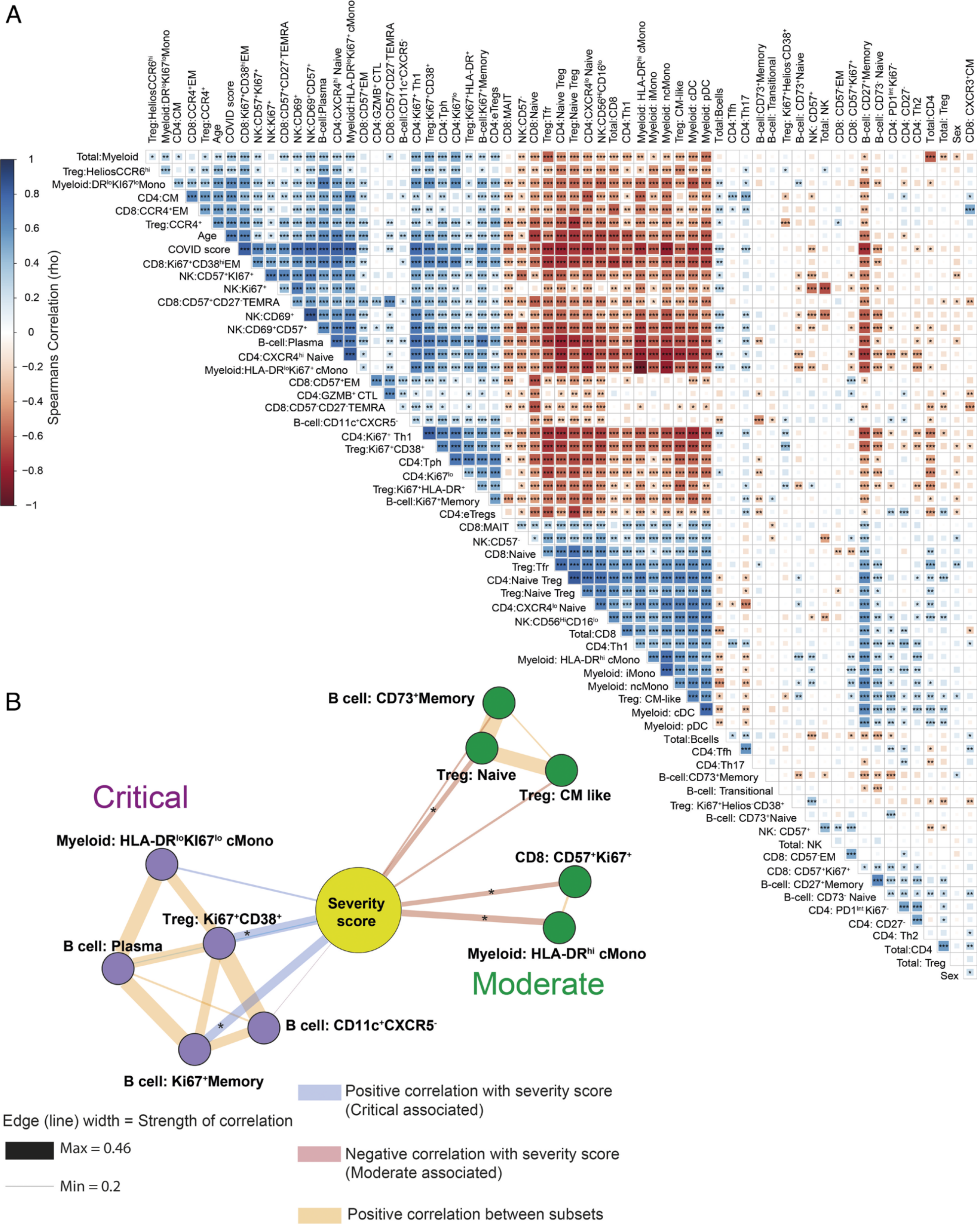
Broad changes to cellular networks in COVID-19. (*A*) Spearman correlations of cellular subset frequencies between indicated subsets, COVID score (1 = healthy, 2 = COVID-19 patient) and sex (1 = male, 2= female). (*B*) Top 5 correlations of subset frequencies with severity of infection scored as WHO ordinal scale (4 = moderate, 6= severe, 7 = critical). Edge width is proportional to the Spearman’s correlation strength. Edges connecting to other cellular subsets are positive correlations between indicated subsets. Layout by ForceAtlas2 using edge weights as input. Significance *P ≤ 0.05, **P ≤ 0.01, ***P ≤ 0.001 by Spearman’s rank correlation (*A* and *B*). Effector memory (EM), CM, terminal effector CD45RA positive (TEMRA). Expression values are arcsinh-transformed (cofactor: 5) dual counts.

We also performed reclustering of NK, CD8, B cells, and myeloid cells and were able to replicate key findings from previous literature, demonstrating the accuracy of this analysis. Severe COVID-19 infection led to increased proportions of activated, proliferating, and cytotoxic CD57^+^CD69^+^ and Ki67^+^ NK cells, while granzyme-B^lo^CD57^–^ NK and CD56^hi^CD16^lo^ NK were decreased (*SI Appendix*, Fig. S3 *A*–*E*) (35). In CD8 T cells, naïve and CD161^+^CCR6^+^ mucosal associated innate T cells were greatly reduced, while several subgroups of proliferating Ki67^+^ EM-like cells characterized by high expression of both CD38 and HLA-DR were increased (*SI Appendix*, Fig. S4 *A*–*E*) (36, 37). Further analysis of the myeloid compartment confirmed the presence of the HLA-DRlo classical Monocytes and loss of intermediate and non-classical Monocyte that several groups have found are associated with severe COVID-19 (*SI Appendix*, Fig. S5 *A*–*E*) (7, 8, 38). The B cell compartment was characterized by a large increase in plasma cells alongside the expansion of rare proliferating memory cells and CD11c^+^CXCR5^–^ atypical B cells similar to those observed by Woodruff et al. (39), while nonproliferating memory B cells were generally reduced (*SI Appendix*, Fig. S6 *A*–*E*). Since we collected information on many cell types, we next sought to use this information to determine the associations between these changing cellular populations by correlation analysis. To avoid undue influence from larger populations, such as cMono, we used subset frequencies within each level of clustering rather than as a proportion of all cells. For example, “Treg: naïve” is the proportion of the naïve Tregs of all Tregs. A large network of cells correlating with each other was increased in COVID-19, including groups of proliferating or activated CD4, CD8, Treg, B cells, plasma cells, NK (CD69^+^), and cMono. (Fig. 4*A*). Highly proliferating Tregs were seen to be in close correlation with plasma cells, HLA-DR^lo^ cMono, and Ki67^+^Th1 cells. Cellular groups that were decreased in COVID-19 patients included several types of less activated Tregs (cTfr, naïve, and CM-like) in correlation with groups of DCs (pDC and cDC), CXCR4^lo^ naïve CD4, naïve CD8, CD56^hi^CD16^lo^ NK cells, and monocyte subgroups (intermediate, nonclassical, and HLA-DR^hi^ cMono). Overall, these results suggest a general shift to dysfunctional and suppressive phenotypes characterized by loss of HLA-DR on monocytes and high levels of suppressive molecules such as CD38 and CTLA-4 expressed by hyperactivated CD4, CD8, and Tregs (Fig. 4*A*). While this analysis revealed broad differences between healthy and COVID-19 patients, it could not clearly show differences within patient subgroups (moderate, severe, and critical patients). To further examine this, we used the same approach but excluded healthy and follow-up samples and restricted visualization of the correlation matrix (*SI Appendix*, Fig. S7*A*) to the top 5 correlations with moderate and critical patients and the level of positive correlation between these cell types. Moderate patients were associated with several populations that were also correlated with HCs such as naïve Tregs, CD73^+^ memory B cells, and HLA-DR^hi^ cMono (Fig. 4*B*). This likely reflects that the moderate patients are at an intermediate phenotype between HCs and more severe patient groups. Upon examination of associations with the critical patient group, we saw that CD38^+^Ki67^+^ Tregs appear to be a central hub around which dysfunctional HLA-DR^lo^Ki67^lo^ cMono, plasma cells, CD11c^+^CXCR5^–^ B cells, and Ki67^+^ proliferating memory B cells were organized (Fig. 4*B*). To further determine whether we had found key differences in our patient cohort, we also performed principal component analysis (PCA) of the log2 normalized cellular percentages (*SI Appendix*, Fig. S8 *A*–*D*). We found that while PCA clearly separated HCs from COVID-19 patients (*SI Appendix*, Fig. S8 *A* and *B*), it was not able to separate the moderate, severe, and critical patient subgroups (*SI Appendix*, Fig. S8 *C* and *D*). Nevertheless, irrespective of including healthy and follow-up samples in the PCA, CD38^+^Ki67^+^ Tregs as well as cTfr and plasma cells/blasts were among the top 10 loadings for principal component 1, thus confirming these subsets as important contributors to COVID-19 immunobiology.

### Sex-Associated Disruption of Tfr, Tfh, and Plasma Cell/Blast Ratios.

**Fig. 5.**
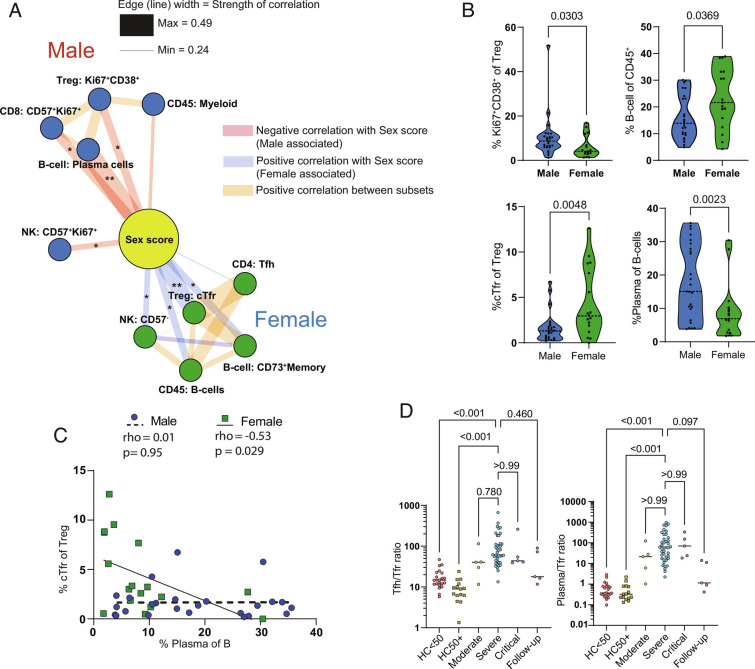
Sex correlations with cellular phenotypes in COVID-19. (*A*) Top 5 cellular correlations with male and female sex in severe COVID-19 patients. Edge width is proportional to correlation strength. Edges connecting to other cellular subsets are positive correlations between indicated subsets. Layout by ForceAtlas2 using edge weights as input. (*B*) Violin plots of sex-specific differences of indicated cellular populations in severe COVID-19 patients. (*C*) Correlation of cTfr and plasma cell frequencies in male and female severe COVID-19 patients. (*D*) Tfh/cTfr ratio and plasma cell/cTfr ratio in indicated patient groups. Correlations in *A* and *C* are Spearman’s rank. Significance by Mann–Whitney test (*B*) or Spearman (*C*). One female patient with undetectable cTfr was excluded from the ratio analysis. Expression values are arcsinh-transformed (cofactor: 5) dual counts.

Given the known importance of sex in susceptibility to COVID-19 (2, 40), we then sought to further dissect the associations of sex in our cohort. Since we were aware of sex bias in the critical patient group (Table 1), we restricted correlation analysis only to the severe patient group (*SI Appendix*, Fig. S7*B*) to avoid confounding effects. Female patients showed an overall increase in the proportion of B cells (Fig. 5*A*), as also seen by Takahashi et al. (41). Plasma cells and Ki67^+^CD38^+^ Tregs were associated with male patients, while the top correlation with female sex was the proportion of cTfr (Fig. 5 *A* and *B*). Since a primary role of Tfr is the control of plasma cell formation (15, 16), this suggested a causative link between the inverse relationship of Tfr and plasma cells/blasts among the sexes. Upon further examination, we found that the proportion of cTfr was reduced as a proportion of Tregs in males (Fig. 5*B*). In line with this, plasma cells were significantly reduced in females compared to males (Fig. 5*B*). We also observed significant negative correlations between Tfr and plasma cells as well as Tfr and CD11c^+^CXCR5^–^ B cells within severe patients (*SI Appendix*, Fig. S7*B*). Further division by sex demonstrated that the negative correlation of Tfr and plasma cells was significant in female but not in male patients (Fig. 5*C*).

While the loss of Tfr is associated with dysregulated control of antibody responses, we also noted a positive correlation between cTfr and Tfh proportions (Fig. 5*A*). Previous studies have demonstrated that the balance between Tfh/cTfr and plasma cell/cTfr is more predictive of dysregulated antibody responses than their individual proportions (30, 42). We also examined these ratios in COVID-19 patients more generally and found that their disruption is apparent in almost all moderate, severe, and critical COVID-19 patients (Fig. 5*D*).

**Fig. 6.**
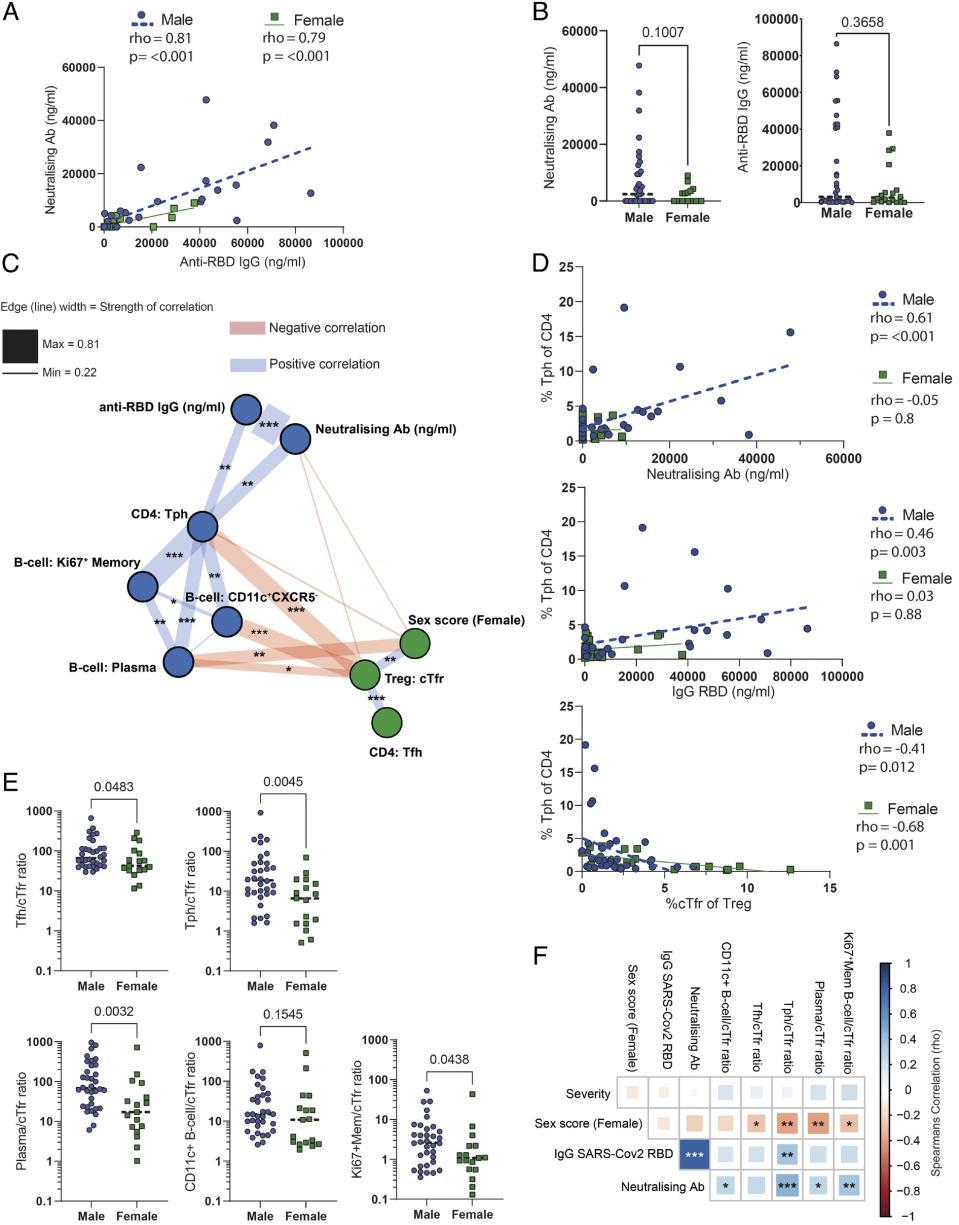
A network of extrafollicular-associated cells is connected with neutralizing antibody responses in male patients and counterbalanced by cTfr. (*A*) Correlation of serum anti-SARS-CoV-2 spike RBD human IgG and neutralizing antibodies in male and female COVID-19 patients measured by ELISA and cytometric bead array, respectively. (*B*) Neutralizing and anti-RBD antibody concentrations in male and female COVID-19 patients. (*C*) Correlation network of antibody production-associated cell types and serum antibodies. (*D*) Correlation between Tph and anti-RBD antibodies, neutralizing antibodies, or cTfr. (*E*) Sex-biased ratios of cTfr and other antibody production-associated cells. (*F*) Matrix of correlations between cTfr antibody production-associated cells and serum antibody levels.

To further clarify the link between cellular populations, patient sex, and antibody responses, we then performed an analysis of serum antibody levels from the same patient cohort. Anti-SARS-CoV-2 RBD IgG and neutralizing antibody levels were strongly correlated with one another and showed a trend to higher levels in male patients, although this was not significant (Fig. 6 *A* and *B*). Correlation of antibody levels with populations of CD4 and B cells revealed that the primary cellular correlation was with PD1^hi^CXCR5^-^CD38^+^HLA-DR^+^CTLA4^lo^ cells that we annotated as Tph cells (Fig. 6 *C* and *D*). Interestingly, these cells formed a male sex-biased hub with additional strong correlations to plasma cells, proliferating (Ki67^+^) memory B cells, and CD11c^+^CXCR5^−^ extrafollicular/atypical B cells (Fig. 6*C*), of which the latter have previously been suggested to be driven by Tph in the context of autoimmunity (43) and appear to be a major source of neutralizing antibodies in COVID-19 (39). Another cluster of PD1^hi^CXCR5^-^ T cells that we annotated as Ki67^+^Th1 could also potentially have been identified as Tph, but the high expression of CTLA-4 and lack of any positive correlation with antibody production (Spearman’s rho −0.2, *P* > 0.1 and rho −0.1, *P *> 0.1, for anti-RBD and neutralizing antibodies, respectively) argue against this. In line with their suppressive function, cTfr were negatively correlated with Tph in both males and females (Fig. 6 *C* and *D*). Since ratios of cTfr against Tfh have previously been shown to be more effective than their individual frequencies at predicting antibody levels (30, 42), we performed further analysis of cTfr ratios. Ratios of Tph, proliferating memory B cells, Tfh, or plasma cells to cTfr showed significant sex bias (Fig. 6*E*). While only Tph individually had a statistically significant correlation with antibody levels, the ratios of all members of the extrafollicular-associated network to cTfr were significantly associated with neutralizing antibody concentrations (Fig. 6*F*). These findings suggest that disruption of cTfr function is a general factor in COVID-19 that is further exaggerated in male patients. In contrast, we did not see significant differences in the proportion of plasma cells/blasts, cTfr, Tph, or CD38^+^Ki67^+^ Tregs between male and female healthy donors (*SI Appendix*, Fig. S8*E*).

In summary, analysis of this cohort of COVID-19 patients has pinpointed potentially crucial roles that Tregs and particularly cTfr may play in organizing multiple aspects of the immune response in COVID-19.

## Discussion

Using mass cytometry to measure broad changes to cellular phenotypes in a large COVID-19 patient cohort, we found a great number of changes, which recapitulated the findings of a number of papers, including abnormal monocytes and highly activated NK and CD8 cells, acting as both confirmation of previous results and demonstration of the accuracy of our analysis (7, 8, 35–38). We also closely examined the status of Tregs during COVID-19. Several papers have observed changes to Treg during COVID-19 (9) variously reporting increases, decreases, or no change in Treg frequency. Alongside differences in patient cohorts, a possible cause of this variability is that not all studies use Foxp3 as part of their identification strategy and instead rely on CD127 and CD25. Since CD25 upregulation and CD127 downregulation are relatively common in highly activated non-Tregs, we would urge caution in reliance on surface markers alone to identify Tregs in the blood of patients with COVID-19. Additionally, Foxp3 alone is not always sufficient to correctly identify Tregs (25), suggesting that a range of markers and higher dimensional approaches (such as UMAP) are needed to fully separate Tregs from non-Tregs in the context of the aggressive cellular activation seen in COVID-19 patients. In our hands, we did not see a clear change in overall Treg frequency, but in agreement with several other studies, a shift from naïve to effector Tregs was observed (10, 44). We confirm this broad point of a general shift in activation and extend these findings by providing further depth of analysis of Treg subpopulations.

A wide range of markers have been used to define various and often overlapping definitions of effector Tregs (28). Here, we show that while there is a general increase in activated and proliferating Treg populations, the CD38^hi^HLA-DR^lo^Ki67^+^ subpopulation showed a clear stepwise increase in frequency between moderate, severe, and critical patients. CD38^lo^HLA-DR^hi^KI67^+^Tregs were also increased in all patient groups but lacked a clear association with severity. HLA-DR^hi^ Tregs are a known population with high suppressive activity (34), whereas CD38^hi^ Tregs are a population that have been studied most often in the context of multiple myeloma (45, 46). Furthermore, CD38^hi^ Tregs from either myeloma patients or healthy donors have increased suppressive function, and CD38-blocking antibodies are able to reduce their function (45, 46). In the context of COVID-19, CD38 expression is widely induced across CD8, CD4, Treg, and plasma cells, suggesting that CD38 expression across these disparate populations may either be driven by a central factor or interaction between these cell types. In addition to Tregs themselves, we also noted an expansion of CTLA-4^hi^ proliferating T cells, particularly in critical patients. CTLA-4 is usually expressed at higher levels in Tregs, where it suppresses the activity of T helper cells (47). CTLA-4 expression by non-Tregs has also been associated with expression of other exhaustion-associated markers such as CD38, PD1, granzyme-B, ICOS, and loss of TCF-1 (48). This was also the case for the CTLA-4^hi^ proliferating T cells in the current cohort, suggesting some level of exhaustion; however, retention of CD27 and their proliferating status argues against this. Several groups have noted increased expression of CTLA-4 either in total or SARS-CoV-2-specific CD4 T cells (5, 6). Here, we noted disruption of usual CTLA-4 expression patterns, with the Ki67^+^Th1 group expressing extremely high levels of CTLA-4 expression, above that of Tregs. This high expression of CTLA-4 on proliferating T cells might, in combination with expansion of effector Tregs, be partly responsible for the establishment of a dysfunctional immune environment characterized by populations such as HLA-DR^lo^ monocytes.

While male sex is a known risk factor for COVID-19 disease outcome, male patients also have increased antibody responses (22). Interestingly, this seems to apply to both neutralizing (21, 49) and autoantibodies (18), suggesting a highly active but dysregulated antibody response. A possible explanation for this phenomenon is the male patient cellular network of PD1^hi^CXCR5^−^ Tph, CD11c^+^ B cells, and plasma cells that was closely linked to neutralizing antibody concentrations in this study. Tph share similar B cell helper functions as Tfh but localize outside the B cell follicle. Due to their recent discovery (50), the function of Tph in viral infections is not well understood, but an association with CD21^low^ B cells has been observed (51). In autoimmunity, Tph have been demonstrated to have a strong correlation with CD11c^+^ atypical B cells in patients with SLE and RA (43, 52). In turn, extrafollicular/atypical B cells have been identified as a key source of early neutralizing antibodies in the context of COVID-19 (39). Together, this suggests that the Tph-driven extrafollicular B cell response may be the source of neutralizing antibodies in this context. In contrast to this male-associated network, we found that cTfr cells were more frequent in female patients and were strongly negatively correlated with Tph, CD11c^+^ B cells, and plasma cells. The Treg subset cTfr has been demonstrated to control antibody production in mice and humans in several contexts. Reduced cTfr or an increased ratio of Tfh to cTfr is associated with autoantibodies and the frequency of plasma cells in autoimmune patients (53–56). In the context of viral infections, Tfr have a fine-tuned role and have been demonstrated to control both self- and foreign reactive antibody responses and quality with a particularly notable effect on plasma cell frequency in murine influenza (15, 16). Accordingly, there is significant evidence that Tfr have an important role in suppressing plasma cell generation, controlling the specificity and memory of the antibody response to acute viral infections, and preventing autoreactive antibodies from developing in this same context. The level of interaction between Tfr, Tph, and CD11c^+^CXCR5^–^ B cells is not well established at this time; however, many Tfr are localized at the T-B border (57), making them well placed to prevent the initial T cell-dependent priming of extrafollicular responses and induction of B cell class switching at this site (58–60). Even though it was sex biased, we still saw that cTfr were reduced in all patients in keeping with another report (61). There is also evidence that this disruption is prolonged as COVID-19 convalescent patients have a decreased proportion of cTfr and an increased proportion of activated Tfh (62). We saw no link between Tfh frequency and serum antibodies in this study, possibly related to the suppression of germinal centers in severe acute COVID-19 patients (63). However, Tfh may have a greater role in the later stages of antibody production once excessive cytokine responses have reduced.

The underlying biology behind the sex-biased antibody production in COVID-19 is not currently clear. In many other contexts, female sex is associated with a stronger antibody response and also greater susceptibility to autoimmune diseases such as SLE, partly due to higher expression of X-linked immune genes such as TLR7 and higher type-1 interferon production (64). In COVID-19, these same factors are important for the improved outcomes in female sex, but antibody levels are paradoxically increased in males. Sex hormones may also underly differences in COVID-19 susceptibility, but their relationship with higher antibody production in this context has not yet been established (64). Since estrogen has been demonstrated to increase Treg formation (65), it is possible that this has a role in this context, but a more specific relationship with Tfr has not yet been established.

In summation, in all cases, Treg subgroups were central parts of the top 5 most severity-associated (Ki67^+^CD38^+^: critical COVID-19, naïve Tregs and CM-like Treg: moderate COVID-19) or sex-associated (cTfr: female patients, Ki67^+^CD38^+^: male patients) cellular populations in COVID-19. While further work is required to truly separate cause from effect, the reduction of cTfr in all COVID-19 patients, which is further exaggerated in male patients, may underly dysregulated antibody production driven by Tph and atypical B cell responses.

## Materials and Methods

### Study Design.

PBMC samples were collected from a cohort of COVID-19 patients and HCs (Table 1). We enrolled hospitalized cases diagnosed as COVID-19 by physicians using clinical manifestation and PCR test results. Samples were collected from August 2020 to May 2021 at Osaka University Hospital. Control subjects were collected at Osaka University Graduate School of Medicine and affiliated institutes. Due to their generally lower age, the HC group was split by age groups into those above (HC 50+) and below 50 (HC < 50) years of age to allow closer comparison of COVID-19 patients with similarly age-matched controls. Patients with COVID-19 were grouped by the WHO eight-point ordinal scale for clinical improvement (66), with 4 = moderate (oxygen by mask or nasal prongs), 6 = severe (intubation and mechanical ventilation), and 7 = critical (ventilation + additional organ support – pressors, RRT, ECMO). All participants provided written informed consent. This study was approved by the ethical committees of Osaka University (IRB no. 734-14) and Osaka University Hospital (IRB no. 20118-4).

### Mass Cytometry Antibody Production.

Indium-113 and -115 and gadolinium-157 were obtained from Trace Sciences, and cisplatin-195 and -196 were obtained from BuyIsotope. Indium and lanthanide isotopes were conjugated to antibodies with the MaxPar conjugation kit (X8 polymer), while Cadmium isotopes were conjugated to antibodies with the MaxPar conjugation kit (MCP9 polymer) according to the manufacturer’s instructions. Platinum-labeled antibodies were conjugated with cisplatin as previously described (67). Conjugated antibodies were stored in a PBS-based antibody stabilizer or HRP-protector stabilizer for cadmium labeling (Candor Biosciences). All antibodies were titrated for optimal staining concentrations using control PBMCs.

### CD45 Barcoding and Cell Staining.

A total of 9 separate experiments were performed. In each experiment, frozen PBMC samples stored in gas-phase liquid nitrogen in 1 ml Cellbanker 1 (Takara Bio) were defrosted in a 37 °C water bath for 1-2 minutes, transferred to a 15-ml tube and 5 ml of prewarmed RPMI containing 10% FCS and 20IU/ml Pierce Universal Nuclease for cell lysis was added. Samples were then washed twice with the same buffer and live cells counted by trypan blue exclusion. In each experiment, up to 1.5 × 10^6^ viable cells per sample were labeled with a six choose-two pattern of anti-CD45 barcodes (113In, 115In, 194Pt, 195Pt, 196Pt, and 198Pt) to give a combination of up to 15 barcoded samples per experiment. The samples were incubated with CD45 barcodes together with FC-block and anti-CXCR5 biotin (*SI Appendix*, Table S1*A*) for 30 min at room temperature (RT), and then washed twice in CyFACS buffer (PBS with 0.1% BSA and 2 mM EDTA). The barcoded cells were then pooled and washed once more with CyFACS buffer. Then, the cells were stained with a metal-conjugated surface stain antibody cocktail for 45 min at RT (*SI Appendix*, Table S1*B*). The cells were then washed twice in CyFACS buffer, stained for viability with dichloro-(ethylenediamine) palladium (II) (DCED palladium (*SI Appendix*, Table S1*C*) (68) in PBS for 5 min at RT, washed, and then fixed and permeabilized using the Foxp3 Transcription Factor Staining Buffer Set according to the manufacturer’s protocol (eBioscience). The cells were subsequently stained with a metal-conjugated intracellular antibody cocktail for 45 min at 4 °C (*SI Appendix*, Table S1*D*) and then washed twice in CyFACS buffer and once in PBS. The cells were then fixed overnight in 1.6% formaldehyde solution containing DNA Cell-ID Intercalator-103Rh (Fluidigm). While DCED palladium contains approximately 11% 110Pd, we find this does not adversely affect the resolution of 110Cd-based CD3 staining in live cells.

### Mass Cytometry Data Acquisition.

Prior to data acquisition, the cells were washed once in CyFACS buffer and twice in MilliQ H_2_O. The barcoded samples were split into separate tubes of up to 2 × 10^6^ cells, and centrifuged, the supernatant was removed, and samples were left as pellets until shortly before running each tube. The cells were then diluted to 1 × 10^6^ cells/mL in Milli-Q H_2_O containing 15% EQ Four Element Calibration Beads (Fluidigm) and passed through a 35-μm filter immediately before running. The cells were acquired at a rate of 200 to 300 cells/s using a Helios mass cytometer (Fluidigm). Flow Cytometry Standard (FCS) files were normalized to EQ bead signals by the Fluidigm normalizer software.

### Mass Cytometry Data Analysis.

For analysis of the mass cytometry results, gating and debarcoding was performed manually using Cytobank software (Beckman Coulter). Cells were initially gated as live, DNA^+^, and CD45^+^ singlets with normal ion cloud Gaussian parameters. Nine Batch control samples (using two lots of healthy PBMCs) were examined for signs of batch effect and then excluded from further analysis. Three severe COVID-19 samples and two HC samples with low cell recovery <10,000 and a high proportion (>10%) of dead cells by palladium inclusion were removed from the analysis. All other samples had a viability of >90% prior to the removal of dead cells by gating. A maximum of 200,000 cells per sample was used for analysis. Following these data-filtering steps, a median of 100,943 (minimum of 18,000) cells per sample were used with a total dataset size of 10,013,285 live CD45^+^ singlets (Fig. 1*A*).

All dual count data channels were arcsinh transformed (cofactor: 5) and then compensated by the CATALYST R package preprocessing workflow (1.14.0) (69) in R (4.0.3). Analysis of data was primarily performed as in “CyTOF workflow: differential discovery in high-throughput high-dimensional cytometry datasets” version 4 (70) as implemented in the CATALYST R package (1.14.0) with packages cowplot (v1.1.1), flowCore (2.2.0), diffcyt (1.10.0), scater (1.18.3), SingleCellExperiment (1.12.0), and ggplot2 (3.3.3). All cells were clustered by FlowSOM in the CATALYST R package with both x-dim and y-dim set to 10 to provide 100 initial SOM clusters, with the consensus meta-clustering level varying from 50 to 20 in line with the expected complexity of the population. The initial 100 SOM clusters and meta-clustering were then examined manually (by expression heatmaps and UMAP or t-SNE) to find the point at which significant populations of interest were inappropriately merged. The meta-clustering level above this point was selected and used as the basis for manually merging populations to annotated subpopulations with clearer interpretations or dynamics. In all cases, initial analysis was rerun several times with new seeds to confirm that similar populations were being reproducibly found before proceeding to the refinement of the cluster numbers. For in-depth analysis of subpopulations, all cells of a particular group of interest (such as CD4 T cells) from the CD45^+^ dataset were selected and separately processed by the same analysis workflow. CD8 cells were subject to a further round of filtering using TCRα/β and CD56 to separate them from TCRα/β^–^ or CD56^+^ cells presumed to be gamma delta T cells and NKT that initially clustered together with CD8 in the first round of analysis. For each analysis of separate subpopulations (CD4, CD8, B cells, etc.), markers used as the basis for clustering “type markers” (70) were altered to select for those with a clear dynamic range of expression or biological interpretability and maximize signal to noise by exclusion of irrelevant markers. All “type markers” were displayed in expression heatmaps Figs. 2*A* and 3*A* and *SI Appendix*, Figs. S1*A*, S3*A*, S4*A*, S5*A*, and S6*A*). Markers displayed in expression heatmaps were trimmed to the 99% percentile of each marker, scaled, and then aggregated, preserving information about expression differences both between markers and between clusters (70). For dimensionality reduction, samples were downsampled to a maximum of 1,000 cells per sample. UMAP was performed with nearest neighbors set to 15 with the exceptions of the CD8 and NK UMAPs which were set to 20 and 25, respectively, for clearer plotting. Markers displayed in UMAPs were trimmed to the 99% percentile and then scaled. Contours were added by ggplot2 (3.3.3) and RColorBrewer (1.1-2). Differential cluster abundance analysis by edgeR was performed with diffcyt (v1.10.0) (71) as implemented in the CATALYST R package (v1.14.0). Wilcoxon matched-pairs, Mann–Whitney, or Kruskal–Wallis tests were performed in GraphPad prism (9.2). Fold change heatmaps were made in GraphPad prism with output from EdgeR. In all cases, expression values were derived from arcsinh-transformed (cofactor: 5) dual counts. Except when indicated, all samples were used in all analyses to give the following n numbers: HC < 50, n = 24; HC50+, n = 15; moderate, n = 5; severe, n = 43; critical, n = 7; follow-up, n = 5.

### Trajectory Analysis.

Trajectory analysis was done with PAGA tree in dynverse (72, 73) with packages dynwrap (1.2.1), dynplot (1.0.2.9000), dynmethods (1.0.5), dynguidelines (1.0.1), and dynfeature (1.0.0.9000) in R (4.0.3). Prior to analysis, samples were arcsinh transformed (cofactor: 5), downsampled to 500 cells per sample, and trajectory starting point was defined to be in the recent thymic emigrant naïve CD4+ T cell area (CD45RA^hi^, CD31^hi^). All features except lineage markers (namely CD11b, CD3, CD4, CD8, CD14, CD11c, TCR α/β, CD16, CD56, and CD19) were used for the trajectory analysis.

### Correlation Analysis.

Correlation analysis was performed using package corrplot (0.84) and readxl (1.3.1) in R (4.0.3). Correlation analysis was performed using pairwise Spearman’s rank correlation. Significance analysis of correlations used two-sided Spearman’s rank. Line graphs and Spearman’s correlation analysis as shown in Figs. 5*C* and 6 *A* and *D* were performed in GraphPad prism (9.2). Lines are linear correlation.

### Network Diagrams.

Network diagrams of correlations were produced in Gephi software (0.9.2). For the purposes of easier visual display, the negative correlations to the central nodes (COVID score, age, or sex score) were converted to positive values (x*−1). Only positive correlations between cell types were retained. In Figs. 4*B*, 5*A*, and 6*C*, an ego network with a depth of one was used to display populations with a direct connection to the central node. The top 5 positive and negative correlations with the central node were displayed. Layout was performed with ForceAtlas2 (74) with the following settings: Tolerance 1, scaling 25, gravity 1, prevent overlap ON, and edge weigh influence 1. Following layout, edge widths were rescaled to a minimum of 0.1 and a maximum of 8 in each graph for display purposes.

### Antibody Detection.

Serum SARS-CoV-2 neutralizing antibodies were assayed using the LEGENDplex SARS-CoV-2 neutralizing Ab assay (1-plex) (Biolegend) according to the manufacturer’s instructions. Samples were run on an LSR Fortessa flow cytometer (BD), and data were analyzed using the LegendPlex software v. 2022-02-10 (Biolegend).

Serum human IgG antibodies against the SARS-CoV-2 spike receptor binding domain were assayed using the LEGEND MAX SARS-CoV-2 Spike RBD human IgG ELISA kit (Biolegend) according to the manufacturer’s instructions. Signal was detected on an iMark microplate reader (Bio-Rad) and data were analyzed using Excel v. 16.62 (Microsoft) and GraphPad Prism v. 9.3.1 (GraphPad).

### Figure Arrangement.

Final figures were arranged in Adobe Illustrator (26.0.1).

## Supplementary Material

Appendix 01 (PDF)

## Data Availability

Mass cytometry data are uploaded to flow repository ID: FR-FCM-Z4XN. R code is available on request.
