# Supplementary material for: A sex-biased imbalance between Tfr, Tph, and atypical B cells determines antibody responses in COVID-19 patients
Source: Proc Natl Acad Sci U S A. 2023 Jan 20;120(4):e2217902120. doi: 10.1073/pnas.2217902120 (PMC9942838; doi:10.1073/pnas.2217902120)
Supplement: Supplementary file 1 — Appendix 01 (PDF) [file pnas.2217902120.sapp.pdf]

PNAS

Supporting Information for

A sex-biased imbalance between Tfr, Tph and atypical B cells, determines antibody responses in COVID-19 patients

Authors: Jonas Nørskov Søndergaard[1]†, Janyerkye Tulyeu[1]†, Ryuya Edahiro[2,3], Yuya Shirai[2,3], Yuta Yamaguchi[2,4], Teruaki Murakami[2,4], Takayoshi Morita[2,4], Yasuhiro Kato[2,4], Haruhiko Hirata[2], Yoshito Takeda[2], Daisuke Okuzaki[5,6,9,10], Shimon Sakaguchi[7,8*], Atsushi Kumanogoh[2,4,9,10], Yukinori Okada[3,9,10,11,12,13,14], James Badger Wing[1,15*]

†Equal contribution

Affiliations:

[1]Human Immunology Team, Center for Infectious Disease Education and Research (CiDER), Osaka University, Suita, Japan

[2]Department of Respiratory Medicine and Clinical Immunology, Osaka University Graduate School of Medicine, Suita, Japan.

[3]Department of Statistical Genetics, Osaka University Graduate School of Medicine, Suita, Japan.

[4]Department of Immunopathology, Immunology Frontier Research Center (WPI-IFReC), Osaka University, Suita, Japan.

[5]Laboratory of Human Immunology (Single Cell Genomics), WPI-IFReC, Osaka University, Suita, Japan.

[6]Genome Information Research Center, Research Institute for Microbial Diseases, Osaka University, Suita, Japan

[7]Laboratory of Experimental Immunology, WPI-IFReC, Osaka University, Suita, Japan.

[8]Department of Experimental Pathology, Institute for Frontier Medical Sciences, Kyoto University, Kyoto, Japan.

[9]Integrated Frontier Research for Medical Science Division, Institute for Open and Transdisciplinary Research Initiatives (OTRI), Osaka University, Suita, Japan.

[10]CiDER, Osaka University, Suita, Japan.

[11]Laboratory of Statistical Immunology. WPI-IFReC, Osaka University, Suita, Japan.

[12]Integrated Frontier Research for Medical Science Division, Institute for Open and Transdisciplinary Research Initiatives, Osaka University, Suita, Japan.

[13]Laboratory for Systems Genetics, RIKEN Center for Integrative Medical Sciences, Yokohama, Japan.

[14]Department of Genome Informatics, Graduate School of Medicine, the University of Tokyo, Tokyo 113-0033, Japan.

[15]Laboratory of Human Single Cell Immunology, WPI-IFReC, Osaka University, Suita, Japan.

*Shimon Sakaguchi and James Wing

Email: shimon@ifrec.osaka-u.ac.jp or jbwing@ifrec.osaka-u.ac.jp

This PDF file includes:

Figures S1 to S8
Table S1

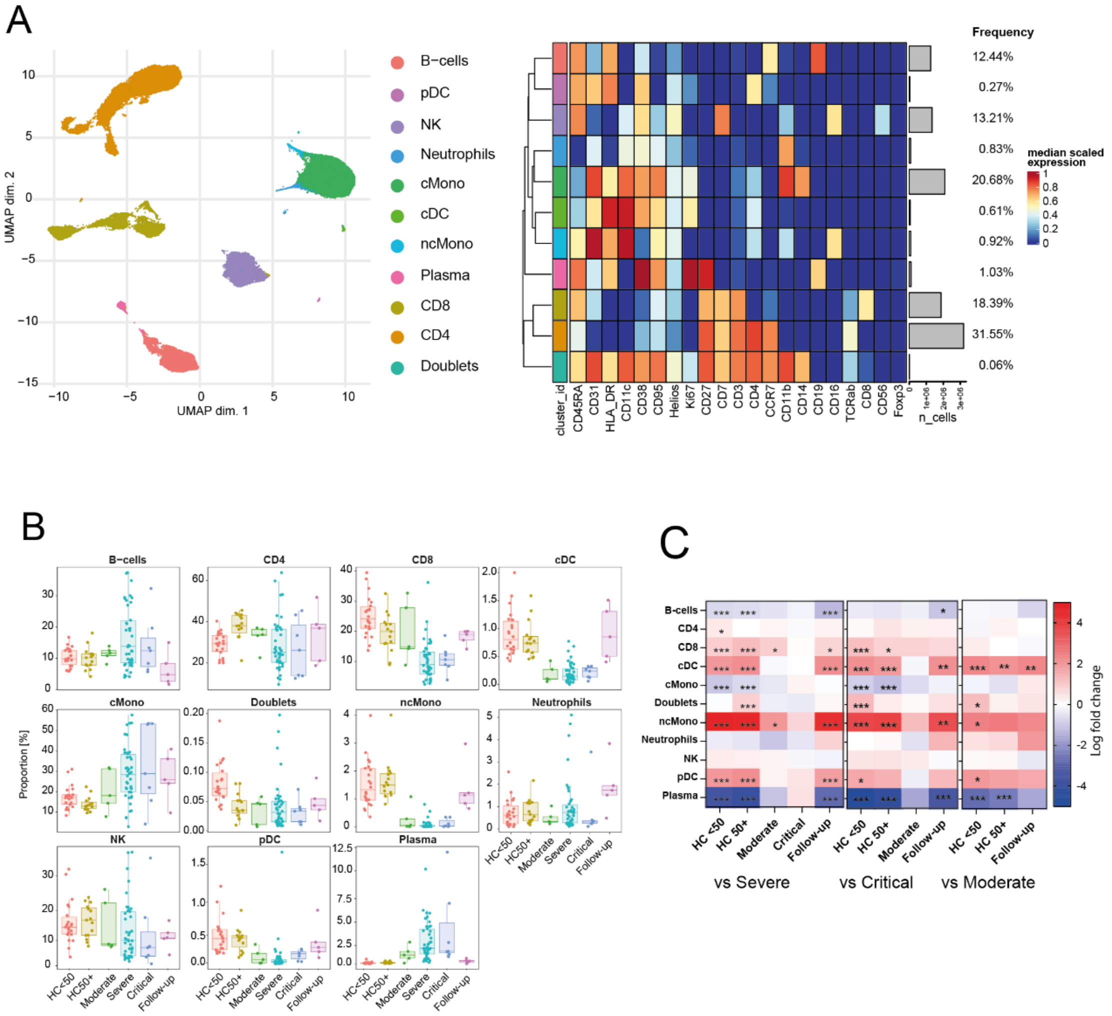

Fig. S1. Alterations to frequency of CD45$^+$ cells. A) UMAP and expression heatmap of FlowSOM clusters from 10,000,000 cells across 100 donors. **B)** Frequency boxplots of proportion of CD45$^+$ cell from indicated clusters. Healthy controls under 50 years of age (HC<50), healthy control of 50 or over (HC50+), moderate, severe, critical or follow-up COVID-19 patients. **C)** Comparison of the fold change (log_2) in cluster-frequency between the indicated group and severe (left), critical (middle), and moderate (right) COVID-19 patients. *p≤0.05, **p≤0.01, ***p≤0.001 by edgeR.

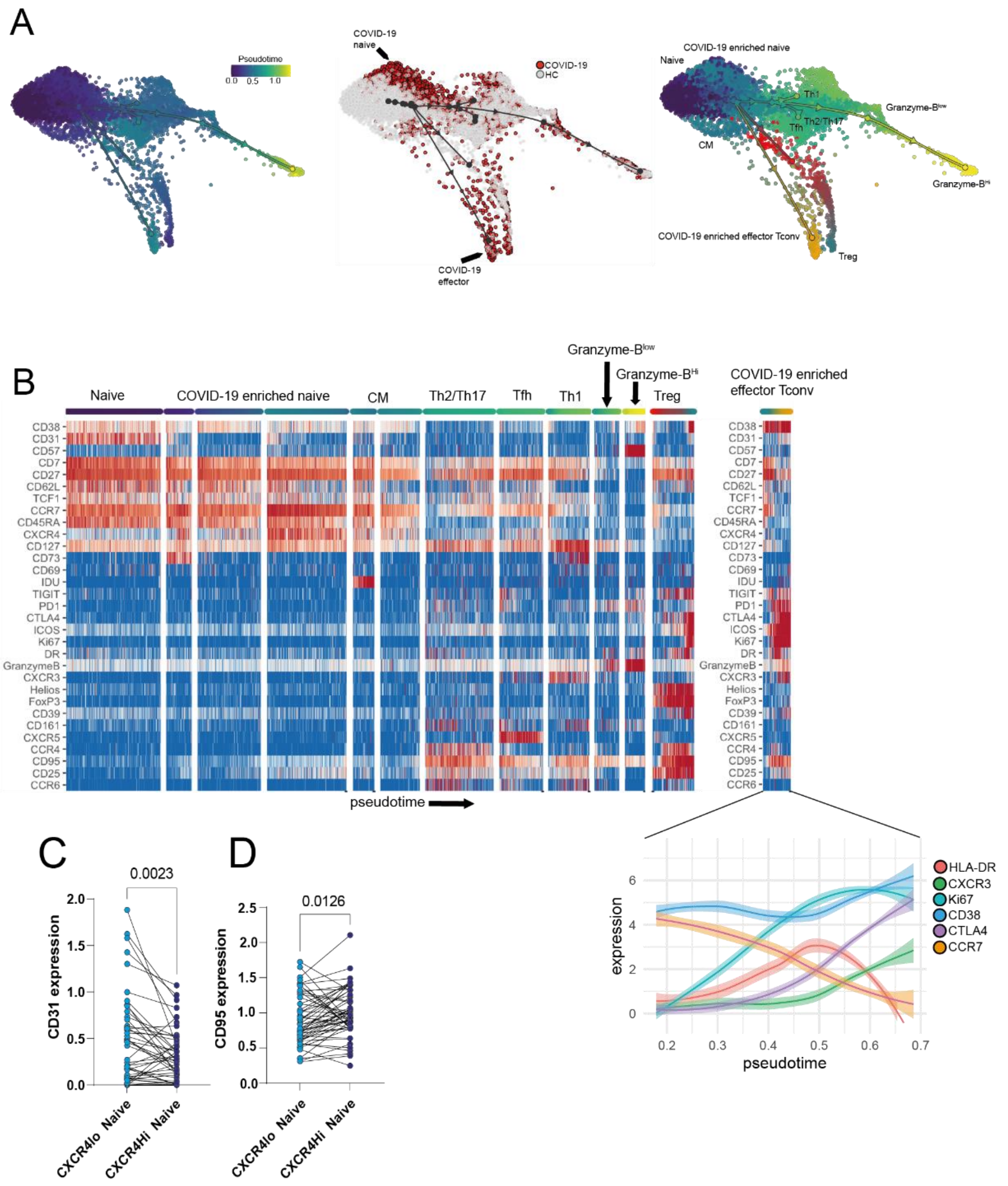

Fig. S2. Trajectory analysis of CD4 T-cells in COVID-19. A) Force-Atlas2 visualization of CD4 T-cell pseudo time (left) and identified milestones corresponding to specific T cell subsets (right). The middle figure indicates single cells originating from COVID-19 patients or healthy controls. **B)** Heatmap of marker expressions within milestones/subsets. **C)** CD31 expression within clusters $CXCR4^{lo}$ naïve and $CXCR4^{hi}$ naïve from Fig. 2A. **D)** CD95 expression within clusters $CXCR4^{lo}$ naïve and $CXCR4^{hi}$ naïve from Fig. 2A. Central memory (CM).

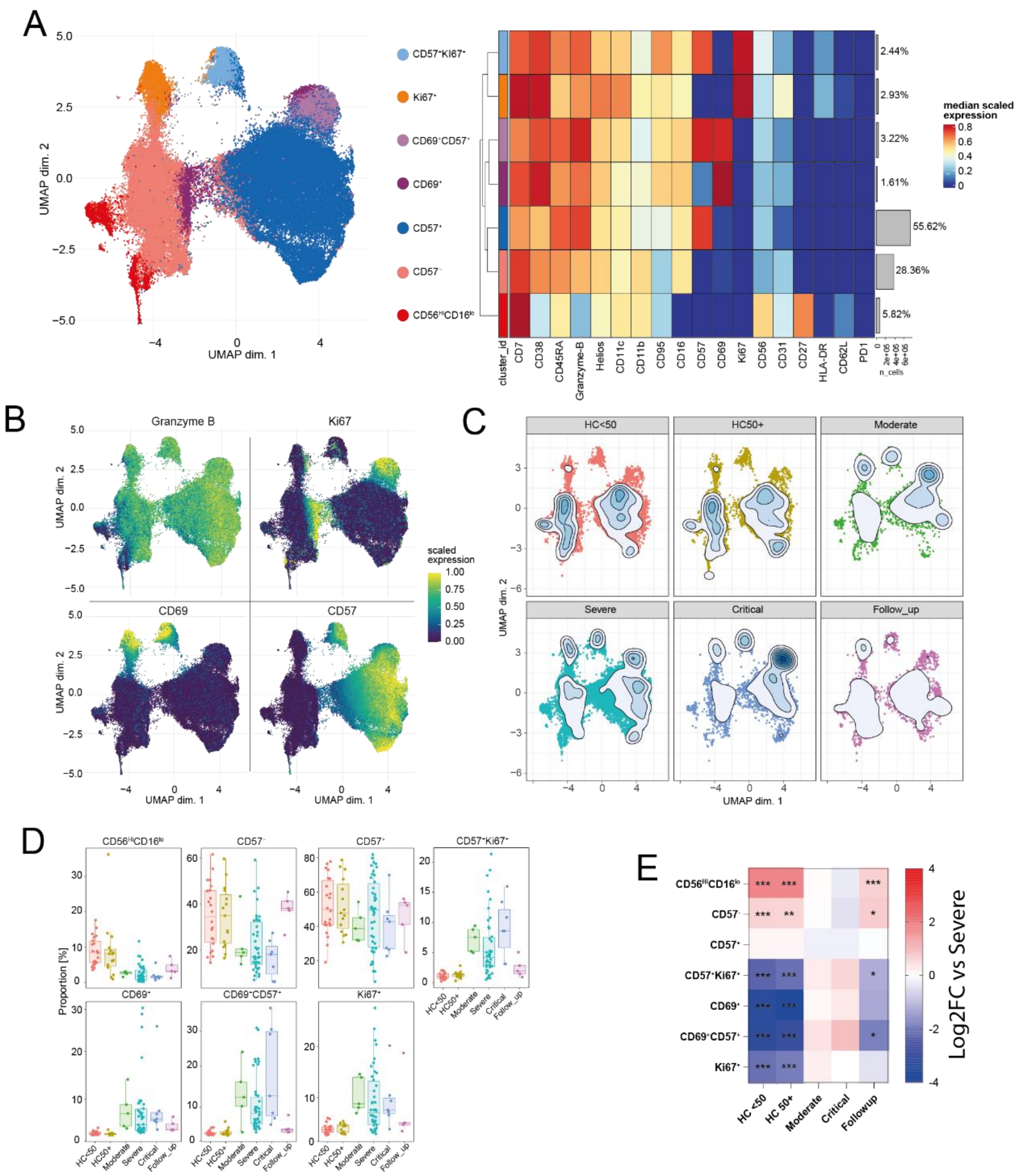

Fig. S3. NK phenotypes in COVID-19. A) UMAP and expression heatmaps of annotated FlowSOM clusters from NK cells (cluster NK from Fig. S1A). **B)** Scaled expression of indicated markers displayed on the UMAP. **C)** Population density of cells displayed on UMAP. **D)** Frequency boxplots of proportion within the NK cell cluster. Healthy controls under 50 years of age (HC<50), healthy control of 50 or over (HC50+), moderate, severe, critical or follow-up COVID-19 patients. **E)** Comparison of the fold change (log_2) in cluster-frequency between the indicated group and severe COVID-19 patients. *p≤0.05, **p≤0.01, ***p≤0.001 by edgeR.

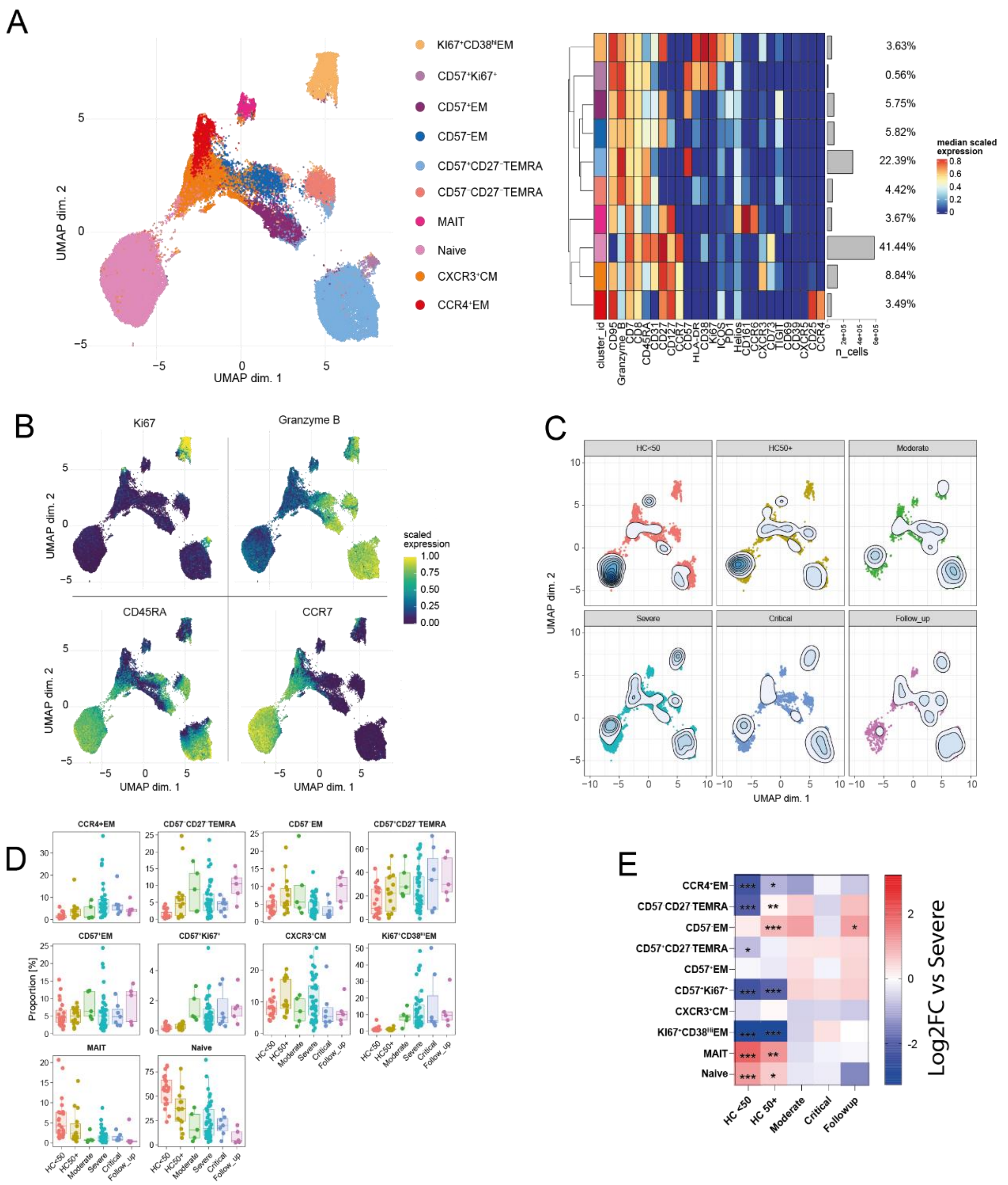

Fig. S4. CD8 phenotypes in COVID-19. A) UMAP and expression heatmaps of annotated FlowSOM clusters from CD8 cells (cluster CD8 from Fig. S1A). **B)** Scaled expression of indicated markers displayed on the UMAP. **C)** Population density of cells displayed on UMAP. **D)** Frequency boxplots of proportion within the CD8^{+} T cells cluster. Healthy controls under 50 years of age (HC<50), healthy control of 50 or over (HC50+), moderate, severe, critical or follow-up COVID-19 patients. **E)** Comparison of the fold change ($\log_2$) in cluster-frequency between the indicated group and severe

COVID-19 patients. *p≤0.05, **p≤0.01, ***p≤0.001 by edgeR. Effector memory (EM), Central memory (CM), Terminal effector CD45RA positive (TEMRA), Mucosal associated invariant T (MAIT).

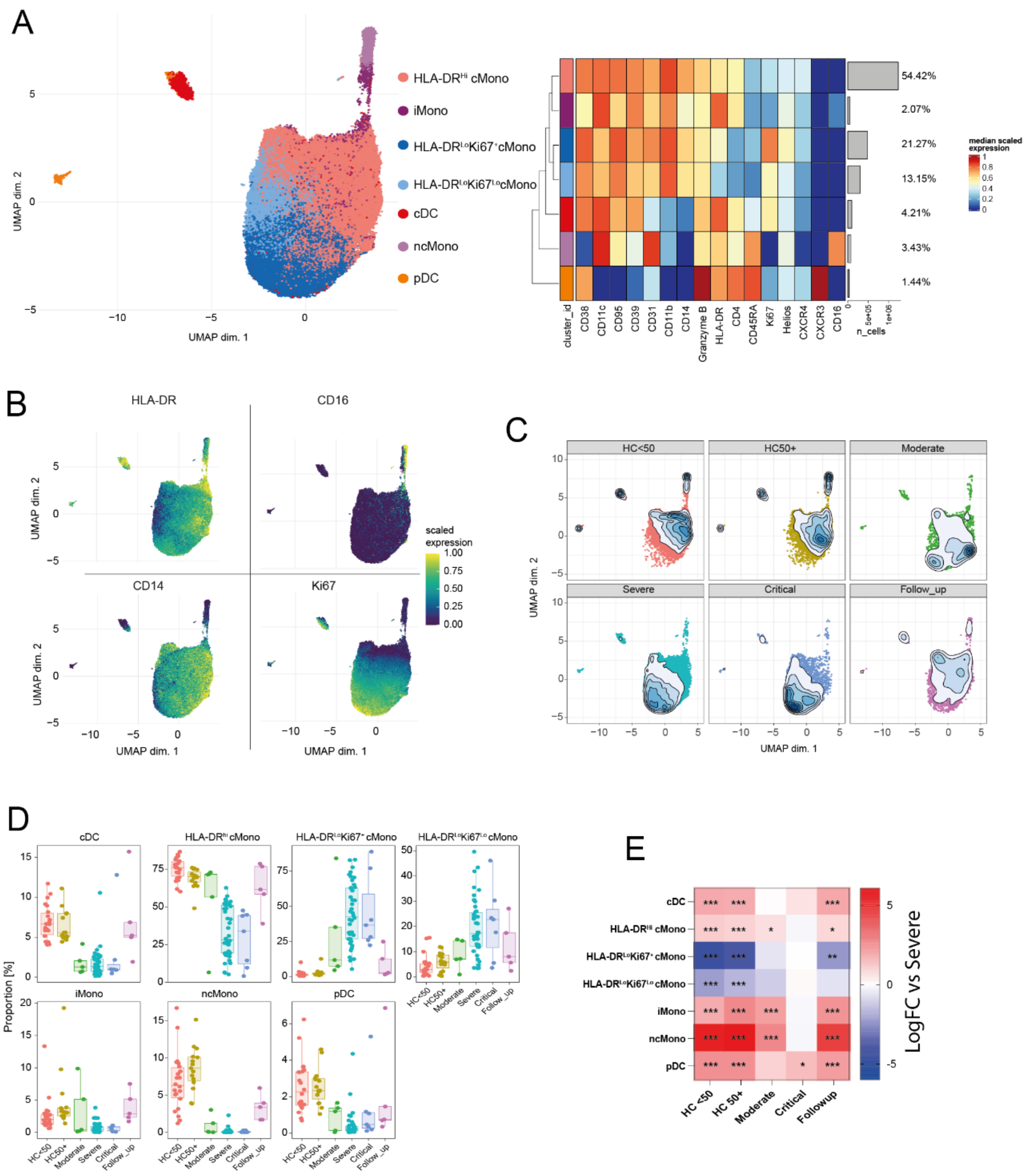

Fig. S5. Myeloid cell and DC phenotypes in COVID-19. A) UMAP and expression heatmaps of annotated FlowSOM clusters from myeloid cells and DCs (clusters cDC, pDC, cMono and ncMono from Fig. S1A) **B)** Scaled expression of indicated markers displayed on the UMAP. **C)** Population density of cells displayed on UMAP. **D)** Frequency boxplots of proportion within the myeloid and DC cluster. Healthy controls under 50 years of age (HC<50), healthy control of 50 or over (HC50+), moderate, severe, critical or follow-up COVID-19 patients. **E)** Comparison of the fold change (log_2) in

cluster-frequency between the indicated group and severe COVID-19 patients. *$p \leq 0.05$, **$p \leq 0.01$, ***$p \leq 0.001$ by edgeR.

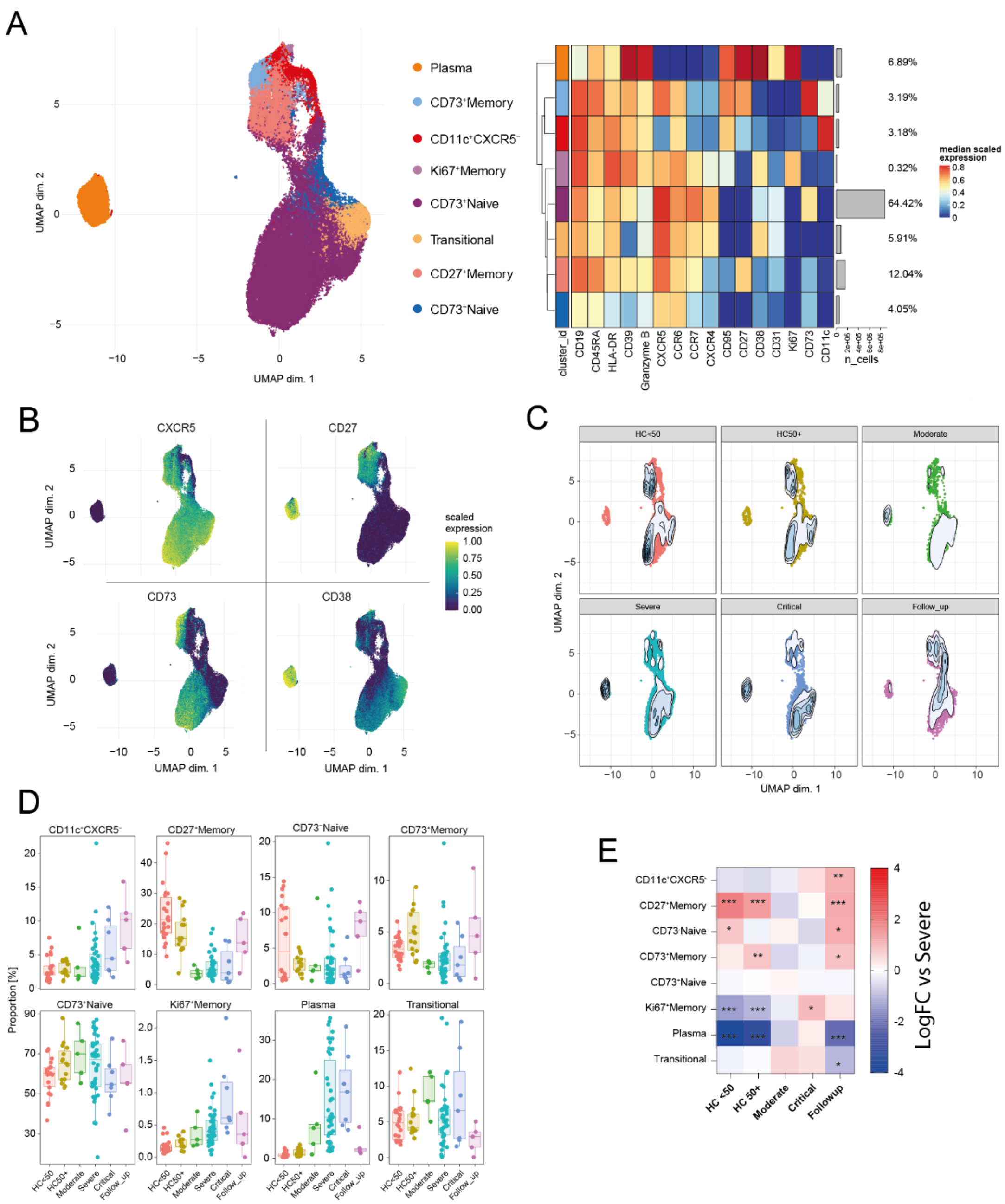

Fig. S6. B-cell and plasma cell phenotypes in COVID-19. A) UMAP and expression heatmaps of annotated FlowSOM clusters from B and plasma cells (clusters "B-cells" and "plasma cells" from Fig. S1A). **B)** Scaled expression of indicated markers displayed on the UMAP. **C)** Population density of cells displayed on UMAP. **D)** Frequency boxplots of proportion within the indicated clusters. Healthy controls under 50 years of age (HC<50), healthy control of 50 or over (HC50+), moderate, severe, critical or follow-

up COVID-19 patients. **E)** Comparison of the fold change (log_2) in cluster-frequency between the indicated group and severe COVID-19 patients. *$p \leq 0.05$, **$p \leq 0.01$, ***$p \leq 0.001$ by edgeR.

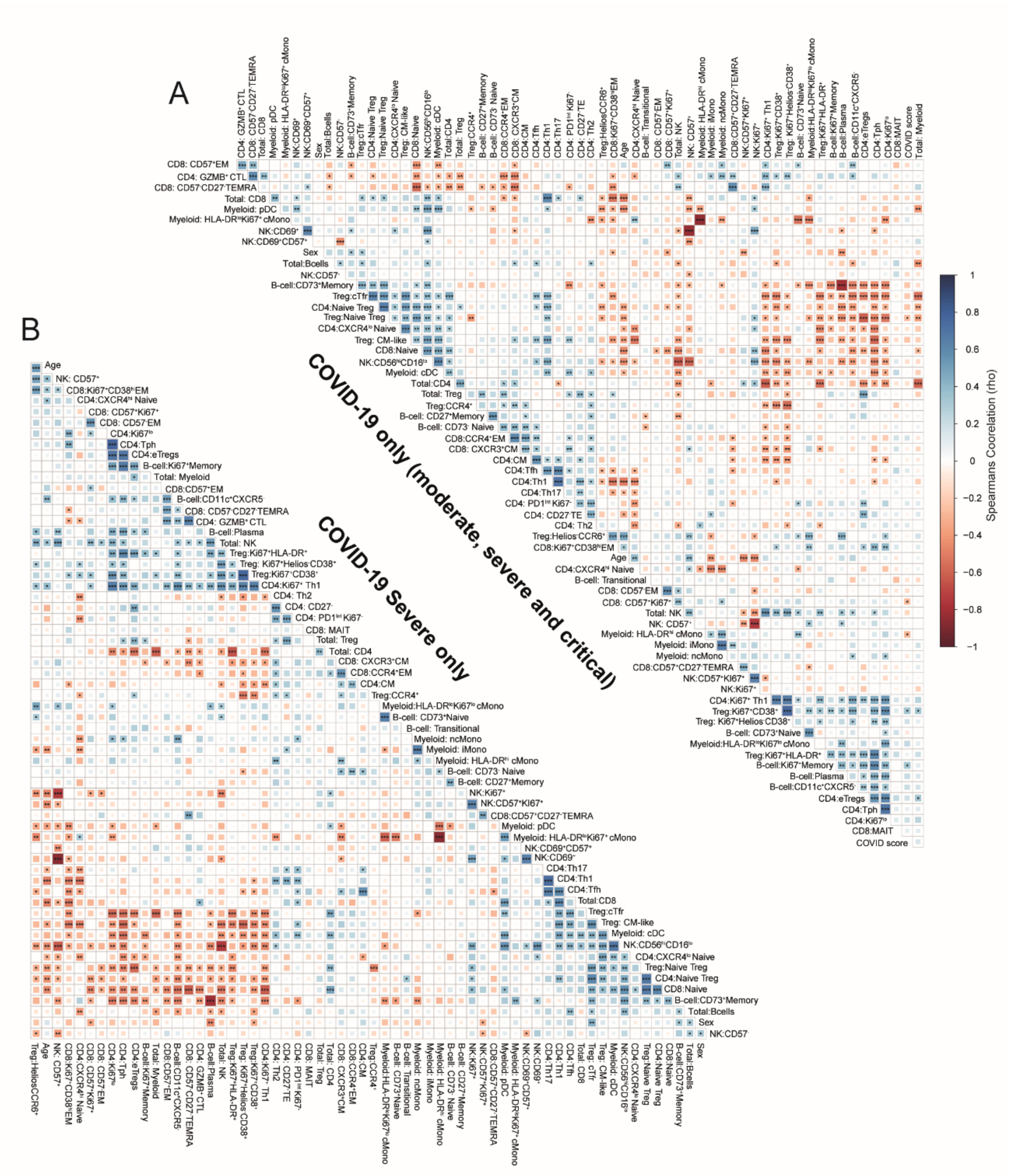

Fig. S7. Cellular correlations in patient subgroups. A) Spearman correlations matrix of subset frequencies from moderate, severe, and critical patient groups with severity of infection scored as WHO ordinal scale (4 = moderate, 6= severe, 7 = Critical) and sex (1 = male, 2= female). **B)** Spearman correlations matrix of subset frequencies from severe COVID-19 patients with severity of infection scored as WHO ordinal scale (4 = moderate, 6= severe, 7 = Critical) and sex (1 = male, 2= female). Correlations by

spearman rank (A, B). Significance *p≤0.05, **p≤0.01, ***p≤0.001. Effector memory (EM), Central memory (CM), Terminal effector CD45RA positive (TEMRA).

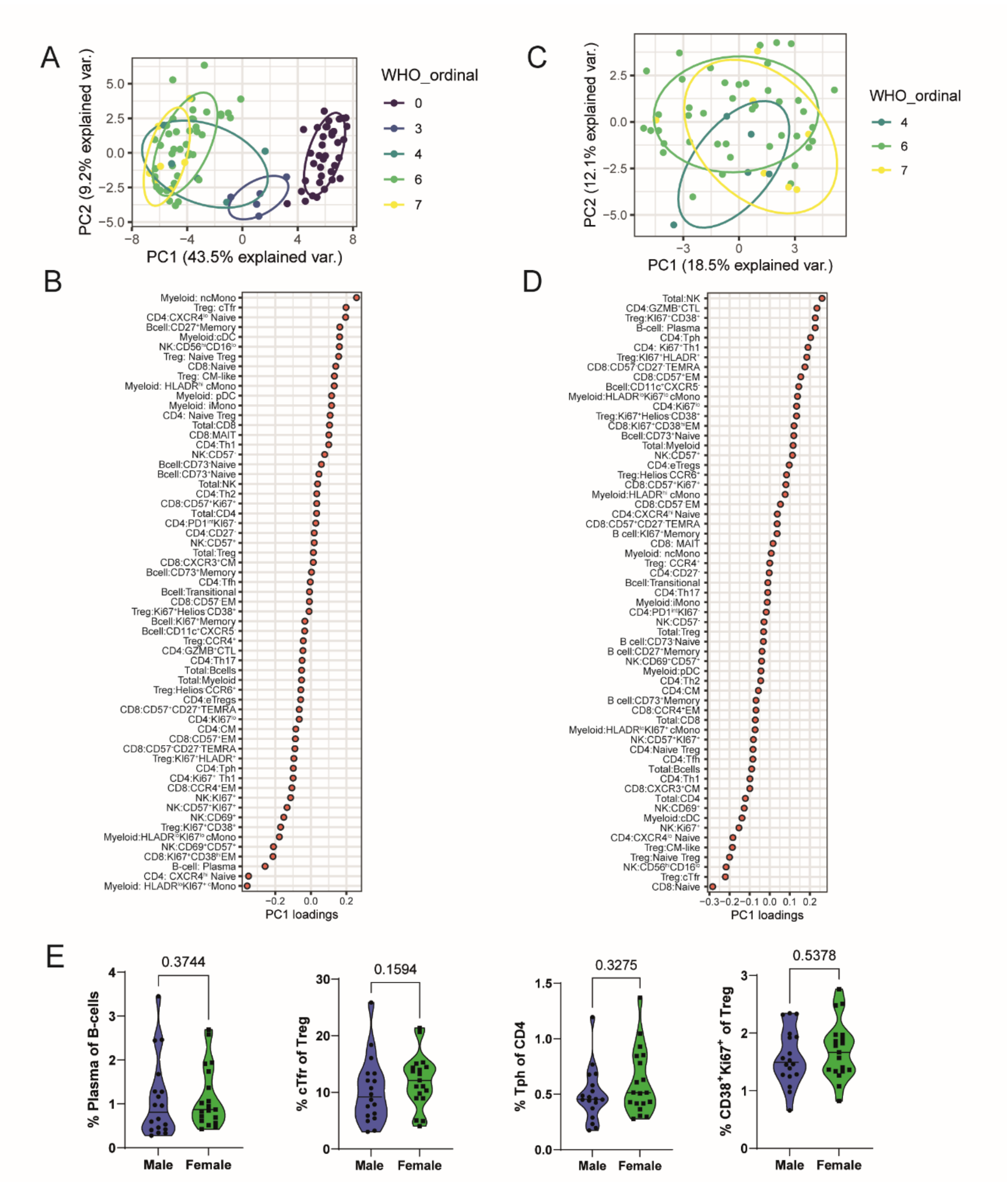

Fig. S8. Principal component analysis and sex differences in healthy controls. A) Factorial map of the principal component (PC) analysis separates log2 normalized cellular fractions of patients classified into WHO ordinal categories of COVID severity. 0 = Healthy donor; 3 = follow-up; 4 = moderate, 6 = severe, 7 = critical. The proportion of

variance explained by each PC is indicated in parenthesis. **B)** PC1 loadings for PCA in A). **C-D)** PCA analysis, but only using WHO ordinal category moderate, severe, and critical. **E)** Violin plots of sex specific differences of indicated cellular populations in healthy controls. Significance by Mann-Whitney test.

Table S1. Mass cytometry staining panel

**Table S1A: Barcodes**				
**Label**	**Target**	**Clone (Product name)**	**Manufacturer**	**Titre**
113In	CD45	HI30	Biolegend	100
115In	CD45	HI30	Biolegend	100
194Pt	CD45	HI30	Biolegend	100
195Pt	CD45	HI30	Biolegend	100
196Pt	CD45	HI30	Biolegend	100
198Pt	CD45	HI30	Biolegend	100
	Fc receptors	(Human TruStain FcX)	Biolegend	50
Biotin	CXCR5	RF8B2	BD	25
**Table S1B: Surface stain**				
**Label**	**Target**	**Clone (Product name)**	**Manufacturer**	**Titre**
110Cd	CD3	UCHT1	Biolegend	50
111Cd	CD4	RPA-T4	Biolegend	100
112Cd	CD8	RPA-T8	Biolegend	50
114Cd	CD14	M5E2	Biolegend	100
127I	Active DNA production	(Cell-ID IdU)	Fluidigm	1000
141Pr	CCR6	G034E3	Fluidigm	50
144Nd	CD7	6B7	Biolegend	100
145Nd	CD56	HCD56	Biolegend	50
147Sm	CD62L	DREG-56	Biolegend	200
148Nd	CD161	HP-3G10	Biolegend	100
149Sm	CCR4	L291H4	Fluidigm	400
151Eu	ICOS	C398.4A	eBioscience	100
152Sm	CD69	FN50	Biolegend	100
153Eu	CD45RA	HI100	Biolegend	100
154Sm	CD73	AD2	Biolegend	100
155Gd	CCR7	G043H7	Biolegend	100
156Gd	CXCR4	12G5	Biolegend	50
157Gd	CD16	3G8	Biolegend	100
158Gd	CD27	L128	Fluidigm	200
159Tb	CD19	HIB19	Biolegend	200
160Gd	CD39	A1	Fluidigm	100
161Dy	CD11c	3.9	Biolegend	100
163Dy	TCR α/β	IP26	Biolegend	100
164Dy	CD95	DX2	Fluidigm	100

165Ho	Biotin	1D4-C5	Fluidigm	50
166Er	PD1	EH12.2H7	Biolegend	100
167Er	CD31	WM59	Biolegend	200
169Tm	CD25	2A3	Fluidigm	100
171Yb	CD57	HCD57	Biolegend	400
172Yb	CD38	HIT2	Fluidigm	100
173Yb	TIGIT	MBSA43	eBioscience	100
174Yb	HLA-DR	L243	Fluidigm	200
175Lu	CXCR3	G025H7	Biolegend	100
176Yb	CD127	A019D5	Fluidigm	100
209Bi	CD11b	ICRF44	Fluidigm	100
**Table S1C: Viability stain**				
**Label**	**Target**	**Product name**	**Manufacturer**	**Titre**
104, 105,106, 108, 110Pd	Dead cells	(Ethylenediamine)palladium (II) chloride >99.99%	Sigma	500 (1µM)
**Table S1D: Intracellular stain**				
**Label**	**Target**	**Clone**	**Manufacturer**	**Titre**
142Nd	Cleaved caspase 3	D3E9	Fluidigm	100
143Nd	TCF1	7F11A10	Biolegend	50
146Nd	Helios	22F6	Biolegend	400
150Nd	Granzyme B	CLB-GB11	Novus	100
162Dy	Foxp3	236A/E7	eBioscience	200
168Er	Ki67	Ki-67	Fluidigm	200
170Er	CTLA-4	14D3	Fluidigm	50
**Table S1E: DNA stain**				
**Label**	**Target**	**Product name**	**Manufacturer**	**Titre**
103Rh	DNA	Cell-ID Intercalator-Rh—500 µM	Fluidigm	500 (1µM)
